# Effects of Dietary Oleacein Treatment on Endothelial Dysfunction and Lupus Nephritis in Balb/C Pristane-Induced Mice

**DOI:** 10.3390/antiox12061303

**Published:** 2023-06-19

**Authors:** Rocío Muñoz-García, Marina Sánchez-Hidalgo, Manuel Alcarranza, María Victoria Vazquéz-Román, María Alvarez de Sotomayor, María Luisa González-Rodríguez, María C. de Andrés, Catalina Alarcón-de-la-Lastra

**Affiliations:** 1 Department of Pharmacology, Faculty of Pharmacy, Universidad de Sevilla, 41012 Seville, Spain; rmgarcia@us.es (R.M.-G.); hidalgosanz@us.es (M.S.-H.); malcarranza@us.es (M.A.); aldesoto@us.es (M.A.d.S.); 2 Instituto de Biomedicina de Sevilla, IBiS/Hospital Universitario Virgen del Rocío/CSIC/Universidad de Sevilla, 41013 Seville, Spain; 3 Department of Normal and Pathological Cytology and Histology, Faculty of Medicine, Universidad de Sevilla, 41012 Seville, Spain; mvazquez2@us.es; 4 Department of Pharmaceutical Technology, Faculty of Pharmacy, Universidad de Sevilla, 41012 Seville, Spain; malugoro@us.es; 5 Unidad de Epigenética, Grupo de Investigación en Reumatología (GIR), Instituto de Investigación Biomédica de A Coruña (INIBIC), Complexo Hospitalario Universitario, de A Coruña (CHUAC), Sergas, 15006 A Coruña, Spain; ma.carmen.de.andres.gonzalez@sergas.es

**Keywords:** endothelial dysfunction, epigenetic, immunomodulation, lupus nephritis, miRNAs, nutritional therapy, oleacein, pristane, systemic lupus erythematosus

## Abstract

Systemic lupus erythematosus (SLE) is a chronic immune-inflammatory disease characterized by multiorgan affectation and lowered self-tolerance. Additionally, epigenetic changes have been described as playing a pivotal role in SLE. This work aims to assess the effects of oleacein (OLA), one of the main extra virgin olive oil secoiridoids, when used to supplement the diet of a murine pristane-induced SLE model. In the study, 12-week-old female BALB/c mice were injected with pristane and fed with an OLA-enriched diet (0.01 % (*w*/*w*)) for 24 weeks. The presence of immune complexes was evaluated by immunohistochemistry and immunofluorescence. Endothelial dysfunction was studied in thoracic aortas. Signaling pathways and oxidative-inflammatory-related mediators were evaluated by Western blotting. Moreover, we studied epigenetic changes such as DNA methyltransferase (DNMT-1) and micro(mi)RNAs expression in renal tissue. Nutritional treatment with OLA reduced the deposition of immune complexes, ameliorating kidney damage. These protective effects could be related to the modulation of mitogen-activated protein kinases, the Janus kinase/signal transducer and transcription activator of transcription, nuclear factor kappa, nuclear-factor-erythroid-2-related factor 2, inflammasome signaling pathways, and the regulation of miRNAs (miRNA-126, miRNA-146a, miRNA-24-3p, and miRNA-123) and DNMT-1 expression. Moreover, the OLA-enriched diet normalized endothelial nitric oxide synthase and nicotinamide adenine dinucleotide phosphate (NADPH) oxidase-1 overexpression. These preliminary results suggest that an OLA-supplemented diet could constitute a new alternative nutraceutical therapy in the management of SLE, supporting this compound as a novel epigenetic modulator of the immunoinflammatory response.

## 1. Introduction

Systemic lupus erythematosus (SLE) is a chronic systemic autoimmune disease characterized by the deposition of immune complexes and autoantibody production, leading to multiorgan damage [1]. Furthermore, it is known to be more prevalent in women of childbearing age. SLE is characterized by the abnormal regulation of innate and adaptive immune responses and impaired levels of T-helper cell (Th) Th1, Th2, and Th17 cytokines [2]. Although the pathogenesis of SLE is unclear, recent studies suggest some possible molecular mechanisms that are involved in SLE immune-inflammatory disease [3]. In fact, the oxidative response is critical for the pathogenesis of SLE; this is coupled with the inflammatory response, which is a key factor regulating the initial aberrant SLE immune response [4]. Su et al. confirmed the relevant role of nuclear transcription factor-kappa B (NF-κB) activation in the pathogenesis of lupus nephritis (LN) [5]. Additionally, as one of the main signaling pathways, the Janus kinase/signal transducer and transcription activator of transcription (JAK/STAT) mediates the communication from transmembrane receptors to the nucleus. Recent research on this pathway has focused on inflammatory diseases such as SLE [6].

In addition, recent reports support the regulation of nuclear-factor-erythroid-2-related factor 2 (Nrf-2) as a crucial factor in the pathogenesis of SLE. Aparicio-Soto et al. described an important reduction in Nrf-2 expression, a redox-sensitive transcription factor, in the renal tissue of Balb/c pristane-induced mice, leading to the under-expression of antioxidant enzymes such as heme oxygenase-1 (HO-1) [7]. In addition, Shin et al. described activated intracellular “NOD-like” receptor (NLRP) 3 inflammasomes in SLE patients’ monocytes, leading to interleukin (IL)-1β overproduction [8]. Moreover, activated levels of the NLRP3 inflammasome have been established in kidney tissue and isolated macrophages from BALB/c mice [9,10].

Currently, SLE treatment remains complex due to the biological heterogeneity between patients and the limited number of specific targeted therapies. Thus, currently, nutritional therapy for SLE disease development is recognized as an effective and safe tool; it is useful for the management of chronic immune-mediated pathologies with limited drug treatments, such as SLE. Pocovi-Gerardino et al. conclude that the regular consumption of the Mediterranean diet can ameliorate SLE disease activity, reducing cardiovascular risk and other comorbidities [11]. In this context, the intake of extra virgin olive oil (EVOO), the principal source of fat in the Mediterranean diet, is related to the improvement of SLE symptoms due to its antioxidant and anti-inflammatory activities [12].

The beneficial health effects of extra virgin olive oil (EVOO) are known to be due to its fatty acid composition as well as its minority bioactive compounds such as polyphenols, among which are the secoiridoids (ligustroside and oleuropein and their derivatives, oleacein (OLA) and oleocanthal, among others). In the last decade, our research group has demonstrated the therapeutic effects of dietary supplementation with EVOO and its bioactive compounds (hydroxytyrosol, oleuropein, and oleocanthal) in a model of pristane-induced SLE in mice [9,13,14]. This animal model is one of the most commonly used because it simulates the human manifestation of SLE more closely than spontaneous strains [15]. An EVOO-enriched diet prevented the development of lupus nephritis in pristane-induced mice by reducing pro-inflammatory mediators in the kidney and serum and improving the Nrf-2/HO-1 axis in kidney tissue [7]. In addition, different diets enriched with EVOO bioactive compounds, including hydroxytyrosol, oleuropein, and oleocanthal, could regulate JAK-STAT, mitogen-activated protein kinases (MAPKs), and NF-κB signaling pathways and the production of pro-inflammatory biomarkers, leading to attenuated LN [9,13,14].

Recent evidence suggests the cardioprotective and anticancerous activities of OLA in in vitro and in vivo studies [16,17]. Moreover, several in vitro studies demonstrated the anti-inflammatory effects of OLA in immune cells. OLA was able to counteract the acute inflammatory response and oxidative stress induced by bacterial lipopolysaccharide (LPS) in THP-1 cells, leading to a reduction in the expression of pro-inflammatory markers and reactive oxygen species (ROS) production. Moreover, OLA increased the levels of anti-inflammatory cytokines, including IL-10 [18]. Recently, Costa et al. confirmed that OLA could modulate the arachidonic acid-dependent inflammatory cascades and NO levels in RAW 264.7 macrophages [19]. According to these results, we have studied the immunomodulatory effects of OLA, exploring the signaling pathways involved in LPS-activated murine peritoneal macrophages [20]. Furthermore, in the context of immune-inflammatory diseases, OLA was presented as a potent antioxidant and anti-inflammatory in a murine model of encephalomyelitis, improving neuromotor disabilities and reducing central nervous system damage [21]. Although the anti-inflammatory effect of OLA has been demonstrated, its potential as a modulator of the immune-inflammatory response of SLE is still unknown. 

Moreover, epigenetic changes such as DNA methylation, micro(mi)RNA profiling, and histone modifications have been described as playing a pivotal role in the pathogenesis of autoimmune diseases such as SLE [22]. In fact, DNA hypomethylation, altered microRNA(miRNA) expression, and post-translational histone modifications have been described by several authors in SLE patients and murine models [23]. In recent years, epigenetic modifications have been proposed as promising targets for the development of individual therapies with fewer or no side effects. In this context, OLA modulated miRNA-34a and miRNA-155 in adipocytes, counteracting the inflammation via the NF-κB pathway [24], and it was able to regulate the expression of several regulatory microRNAs in melanoma cells [25]. Moreover, OLA promoted the cell cycle and apoptosis in multiple myeloma cells by inhibiting histone deacetylases (HDACs), leading to decreased acetylated histones and gene expression [26]. On the other hand, we recently described the modulation of histone H3 methylation and acetylation in an ex vivo model of LPS-induced peritoneal macrophages [20].

In summary, the present study aimed to evaluate the preventive effects of an OLA-supplemented diet in a murine model of pristane-induced SLE. We analyzed lupus nephritis, conducting histological and biochemical analyses focusing on the molecular mechanisms and signaling pathways involved after dietary treatments. Furthermore, we studied OLA-induced epigenetic changes such as DNA methyltransferase (DNMT-1) and miRNA expression (miRNA-23b, miRNA-24-3p, miRNA-146a, and miRNA-126) in renal tissue and evaluated the effects of the OLA-enriched diet on vascular dysfunction in aortic tissue. 

## 2. Materials and Methods

### 2.1. Reagents

We obtained the following materials from the corresponding suppliers: sucrose pellets (Colorcon Iberica^®^; Barcelona, Spain). Eudragit^®^ EPO (Evonik; Essen, Germany); dichloromethane (DCM), ethanol, ethyl acetate, and hexane (VWR^®^, Madrid, Spain); talc, socium dodecylsulfate, and stearic acid (Acofarma^®^; Madrid, Spain); and Harris’ Haematoxylin, pristane (2, 6, 10, 14-tetramethylpentadecane), titanium dioxide, trypsin, 3,3- diaminobenzidine tetrahydrochloride (DAB) solution, DAPI, Nonident-P40, and casein (Sigma-Aldrich^®^, Burlington, MA, USA). We used the following materials for the immunoprecipitation assays: Cy3-labeled donkey anti-mouse IgG (1:100, Jackson ImmunoResearch Laboratories^®^, West Grove, PA, USA); anti-mouse immunoglobulin(Ig)G (Vector laboratories^®^, Burlingame, CA, USA); anti-rat IgM (Invitrogen^®^, ThermoFisher Scientific, Waltham, MA, USA); Donkey serum (Jackson Immuno-Research Laboratories^®^, West Grove, PA, USA); vectastain ABC-horseradish peroxidase-labeled (HRP) Kit (Vector laboratories^®^, Burlingame, CA, USA). We used the following Western Blot antibodies: anti-rabbit IgG (polyclonal; 1:1000; Cell Signaling Technology^®^ (Danvers, MA, USA); Cat#7074; RRID:AB_2099233), β-anti-mouse IgG (1:2000; polyclonal; Sigma-Aldrich^®^; Cat#A4416; RID:AB_258167), actin antibody (monoclonal; 1:10000; Abcam^®^ (Cambridge, UK); Cat#ab49900; RRID:AB_867494), Ciclooxygenase (COX)-2 (1:1000; monoclonal; Cell Signaling Technology^®^; Cat#12282; RRID:AB_2571729), microsomal prostaglandin E synthase-1 (mPGES-1) (1:1000; polyclonal; Abcam^®^; Cat#ab62050; RRID:AB_2269175), Inducible Nitric Oxide Synthase (iNOS) (1:1000; monoclonal; Cell Signaling Technology^®^; Cat#13120; RRID:AB_2687529), IL-18 (1:1000; polyclonal; Abcam^®^; Cat#ab71495; RRID:AB_1209302), NLRP3 (1:1000; monoclonal; Cell Signaling Technology^®^; Cat#15101; RRID:AB_2722591), Caspase-1 (5:2000; polyclonal; Novus Biologicals^®^ (Littleton, CO, USA); Cat#NBP1-45433; RRID:AB_10008900), adaptor apoptosis-associated speck-like protein containing a caspase recruitment domain (ASC) (1:1000; monoclonal; Cell Signaling Technology^®^; Cat#67824; RRID:AB_2799736), Caspase-11 (1:2000; polyclonal; Novus Biologicals^®^; Cat#NBP1-45453; RRID:AB_10008917), HO-1 (1:1000; polyclonal; Enzo Life Sciences^®^ (Madrid, Spain); Cat#ADI-SPA-896-D; RRID:AB_2039233), pERK (1:1000; monoclonal; Cell Signaling Technology^®^; Cat#4370; RRID:AB_2315112), pP38 (1:1000; monoclonal; Cell Signaling Technology^®^; Cat#4511; RRID:AB_2139682), pJNK (1:1000; polyclonal, Cell Signaling Technology^®^; Cat#4668; RRID:AB_823588), ERK (1:1000; monoclonal; Cell Signaling Technology^®^; Cat#9107; RRID:AB_10695739), P38 (1:1000; monoclonal; Cell Signaling Technology^®^; Cat#8690; RRID:AB_10999090), JNK (1:1000; polyclonal; Cell Signaling Technology^®^; Cat#9252; RRID:AB_2250373), p-endothelial nitric oxide synthase (eNOS) (Thr495) (1:1000; polyclonal; Cell Signaling Technology^®^; Cat#9574; RRID:AB_2153176), eNOS (polyclonal; 1;1000; Cell Signaling Technology^®^; Cat#9572; RRID:AB_329863), nicotinamide adenine dinucleotide phosphate (NADPH) oxidase (NOX)-1 (1:1000; polyclonal; Abcam^®^; Cat#ab131088; RRID:AB_11156101), Nrf-2 (1:1000; monoclonal; Cell Signaling Technology^®^; Cat#12721; RRID:AB_2715528), IκB-α (1:1000; monoclonal; Cell Signaling Technology^®^; Cat#4812; RRID:AB_10694416), NF-κB p50 (1:1000; monoclonal; Cell Signaling Technology^®^; Cat#13586; RRID:AB_2665516), NF-κB p50 (1:1000; monoclonal; Cell Signaling Technology^®^; Cat#8242; RRID:AB_10859369), p- Signal transducer and transcription activator of transcription, (STAT)-3 (1:1000; monoclonal; Cell Signaling Technology^®^; Cat#9145; RRID:AB_2491009), and p-Janus Kinase (JAK)3 (1:1000; monoclonal; Cell Signaling Technology^®^; Cat#5031; RRID:AB_10612243). We also obtained a chemiluminescence substrate (Pierce^®^, Rockford, IL, USA).

### 2.2. Chemical Synthesis of (−)-Oleacein

The starting material, (–)-oleuropein, was purchased from Sigma-Aldrich^®^ (Madrid, Spain). The solvents used for the chromatography separations and synthetic procedures, such as hexane (Hex), ethyl acetate (EtOAc), ethanol (EtOH), methanol (MeOH), dimethyl sulfoxide (DMSO), chloroform (CHCl3), and diethyl ether (Et2O), were purchased from VWR^®^ (Madrid, Spain). The rest of the reagents, solvents, materials, and instruments were described in our previous paper by Montoya et al. [9].

A mixture of (–)-oleuropein (0.40 g) and H_2_O (25 mL) was subjected to microwave irradiation for 13 min (min) at 180 °C. The resulting crude reaction was extracted three times with 30 mL of CHCl3. The organic phases were united, dried over anhydrous Na_2_SO_4_, filtered, and evaporated under reduced pressure, producing a dark brown oil. The dry crude reaction was purified by fast centrifugal partition chromatography (FCPC) (Hex:EtOAc:EtOH:H2O, 1:1:1:1, *v*/*v*/*v*/*v*) to give (–)-oleacein (0.14 g, 58% reaction yield). The structure of (–)-oleacein was confirmed by comparison of the NMR data with those reported in the literature [20,27]. This procedure was repeated several times until obtaining the necessary amount of (–)-oleacein to prepare the dietary treatment.

### 2.3. Diet Elaboration

Diets were enriched with film-coated pellets impregned with OLA (OLA group) or non-impregned film-coated pellets (the naïve and SD-pristane group). At first, OLA (150 mg) was solved in dichloromethane (DCM) (45 mL) and added to 105 g of sucrose pellets, which were placed in a round bottom flask. The DCM was evaporated in a R-200 rotary vacuum evaporator (Büchi^®^, Flawil, Switzerland) for the impregnation of the pellets with OLA. After DCM evaporation, the pellets were dried in the dark, at room temperature.

For film-coating, Eudragit^®^ EPO was used. Eudragit^®^ EPO is an excellent film-forming polymer, which protects the drug from humidity and light. Sodium dodecylsulfate (0.25 g) and stearic acid (0.4 g) were used as a stabilizer and a plasticizer, respectively. Sodium dodecylsulfate and stearic acid were dissolved in absolute ethanol (30 g) for 30 min at 40 °C. Next, 2.65 g of Eudragit^®^ E PO was added and the solution was homogenized with a T18 digital ULTRA-TURRAX^®^ (IKA, Staufen, Germany) over a period of 90 min, forming a colloid. After homogenization, we added titanium dioxide (0.25 g) and talc (0.32 g) and blended it again in the ULTRA-TURRAX^®^ for 15–20 min. Finally, in order to remove particle aggregates, the final solution was passed through a sieve (350 µm). 

Ultimately, 100 g of non-impregnated or OLA-impregnated pellets were coated in a fluidized bed coater system (Glatt^®^, Binzen, Germany) using bottom spray. The atomization pressure (1 bar), air temperature (40 °C), coating solution flow rate (0.7 mL/min), and nozzle diameter (0.5 mm) were kept constant throughout the process. The film-coated pellets were added to standard rodent powder chow (diet supplementation: 0.01% 100 mg/Kg)) and homogenized with a Turbula^®^ 3D mixer (WAB, Basel, Switzerland). Then, compacted diets were prepared based on our previous reports [13].

Experimental diets were prepared following the standard reference diet of the American Institute of Nutrition (AIN). The SLE-pristane and naïve groups were fed with the standard diet (SD) supplemented with non-impregnated film-coated pellets, while the OLA-group mice received a diet enriched with OLA film-coated pellets.

### 2.4. Animals, Diets, and SLE-like Disease Induction

Female BALB/c mice (25 ± 3 g) of 12 weeks of age were supplied by Janvier Labs (Saint-Berthevin Cedex, France). The mice were kept under regulated conditions of humidity of 40–60%, a temperature of 24–25 °C, and a 12 h light/dark cycle. Water and standard rodent chow (Panlab A04^®^, Seville, Spain) were administered ad libitum to animals until the pristane induction of the SLE-like disease. 

When the mice were 16 weeks old, they were randomly separated into three experimental groups: (1) the naïve group, which received a standard diet enriched with non-impregnated film-coated pellets (SD) (*n* = 10); (2) the pristane group (SD-pristane group), which was fed with SD (*n* = 18); and (3) the pristane-oleacein group (OLA), which received a diet enriched with OLA film-coated pellets (0.01% (*w*/*w*)) (*n* = 18). This concentration was selected considering our experience with similar compounds tested in the same experimental mode in previous studies [9,13]. Fresh diets were provided every day; the experimental diets were formulated based on the American Institute of Nutrition standard reference diet and prepared by mixing the pertinent compounds before being stored at −80 °C.

In order to induce SLE-like disease, an intraperitoneal injection of 0.5 mL of pristane (99% pure, Sigma Aldrich^®^ Co., St. Louis, MO, USA) was administered to 16-week-old BALB/c mice (groups 2 and 3) according to the method described by Satoh and Reeves [28]. On the other hand, the animals in the naïve group were injected intraperitoneally with saline solution. The mice were fed fresh diets for 24 weeks. Mortality, weight, and food and water consumption were monitored weekly. All animal care and experimental procedures complied with the guidelines of the European Union regarding animal experimentation (Directive of the European Counsel 2010/630/EU) and followed a protocol approved by the Animals Ethics Committee of the Junta de Andalucía protocol code 23/07/2018/119 and by the Animals Ethics Committee of the University of Seville protocol code CEEA-US2018-11/2.

After 6 months, the mice were euthanized by overdoses of ketamine/xylazine. The spleen and thymus were harvested; kidneys were collected and fixed in fresh formaldehyde for immunohistochemical (IHC), histological, and immunofluorescence (IF) studies; fresh aortas were used for the immediate determination of vascular reactivity; and the remaining renal and vascular tissues were kept at −80 °C.

This in vivo model is used because it simulates the human manifestation of SLE more closely than spontaneous strains [15]. The whole experimental process complies with the ARRIVE guidelines [29]. Appropriate measures were considered to reduce and minimize the number of animals and their suffering. The randomization of animals between groups was carried out in order to generate groups of equal size. 

### 2.5. Kidney Histological Evaluation

Mice kidneys were collected and fixed in formaldehyde. After paraffin embedding, samples were sagittally sectioned at 4 µm thickness. Several sections per animal were routinely stained according to standard staining procedures. General morphological and histopathological changes were assessed by hematoxylin-eosin (H&E); the glomerular status and fibrotic changes were analyzed by periodic acid-Schiff (PAS) and Masson’s trichrome (MT), respectively. All histological studies were performed at the light microscopy level by two different observers who were unaware of the treatment group. Results are expressed as the percentage of cases exhibiting histological changes.

### 2.6. Immunohistochemistry and Immunofluorescence Analysis

To assess the deposition of IgM and IgG immunocomplexes, we used IF and IHC analyses. For immunohistochemical studies, renal silane-coated sections were dewaxed in xylene, and then the sections were rehydrated with decreasing concentrations of alcohol. Subsequently, we performed an antigen retrieval step as described by Yabuki et al. [30]. Initially, sections were incubated with 0.1% trypsin at 37 °C for a period of 30 min (Try-30); next, sections were treated in a heating instrument PTLink (DAKO^®^, Glostrup, Denmark) for 20 min at 96 °C using EnVision Flex Target Retrieval High pH (DAKO^®^, Glostrup, Denmark), according to the manufacturer’s instructions. Subsequently, the samples were treated with 3% hydrogen peroxide for 20 min in order to block endogenous peroxidase activity. Then, they were incubated overnight at 4 °C in a wet chamber, with mouse anti-IgG (Vector laboratories^®^, Burlingame, CA, USA) or rat anti-IgM (Invitrogen^®^, ThermoFisher Scientific, Waltham, MA, USA). As a labeling system, we used the Vectastain ABC-HRP Kit, following the manufacturer’s instructions. The DAB solution was used as a chromogen. Samples were counterstained with Harris’ Haematoxylin, dehydrated, and coverslipped. Finally, photomicrographs were taken using an Olympus photomicroscope and a Nikon DS-Fi3 camera. 

Likewise, we analyzed the deposits of IgM and IgG in renal samples by IF. Kidney sections were deparaffined, hydrated, and pretreated for antigen retrieval following the protocol described previously [30]. Then, in order to block non-specific binding, normal donkey serum (10%) was used for 15 min. The samples were incubated with primary antibodies against either IgG or IgM at 4 °C overnight. After the incubation period and washing, the samples were incubated with Cy3-labeled donkey anti-mouse IgG secondary antibodies for 30 min in a humidified chamber at room temperature. The slides were washed with PBS, 4′,6-diamidino-2-phenylindole (DAPI) (Sigma-Aldrich^®^, Burlington, MA, USA) was added for nuclear counterstaining, and then samples were coverslipped with antifading mounting medium (Mowiol 4–88). Finally, the renal sections were observed under a fluorescence microscope (Olympus BX50) connected to a scientific digital camera (Hamamatsu ORCA-03G).

### 2.7. Western Blotting

Kidney tissues from mice were homogenized in ice-cold lysis buffer (250 mM NaCl, 50 mM Tris–HCl (pH 7.5), 0.5 mM EDTA, 5 mM EG-TA, 8 mM MgCl2, 0.01 mg/mL, pepstatin, 0.01 mg/mL leupeptin, 0.01 mg/mL aprotinin, 1 mM phenylmethylsulfonyl fluoride, using a TissueRuptor (Qiagen^®^, Hilden, Germany) following the methodology described by Aparicio-Soto et al. [7]. The nuclear fraction was also isolated from the kidneys following the method described by Aparicio-Soto et al. [7]. On the other hand, for the homogenization of the thoracic aorta, a radioimmunoprecipitation assay buffer with a micro-pestle motor-driven tissue homogenizer (Heidolph Instruments^®^, Schwabach, Germany) was used. After homogenization, the samples were centrifuged for 10 min at 2000× *g* and 4 °C. The final supernatant was used for protein expression analysis.

After extraction, protein content was measured using a protein assay reagent [31]. Protein samples were separated on a 10% or 15% acrylamide gel by sodium dodecyl sulfate polyacrylamide gel electrophoresis (SDS-PAGE) and transferred to nitrocellulose membranes (BioRad^®^, Hercules, CA, USA). The membranes were blocked for 1–2 h (1% casein and 5% Nonident-P40 in PBS buffer) and incubated overnight at 4 °C with a specific primary antibody. The membranes were then incubated with antirabbit secondary antibody (Cell Signaling^®^, Danvers, MA, USA) or antimouse HRP secondary antibody (Dako^®^, Atlanta, GA, USA). Protein bands were captured using an Amersham Imager 600 (GE Healthcare^®^, Chicago, IL, USA). and analyzed using Image Processing and Analysis in Java (Image J^®^, Bethesda, MD, USA). Measurements were normalized to relative internal controls, as indicated in the legends of the figures. Anti-β-actin, -ERK, -P38, or -JNK were used as loading controls.

### 2.8. Vascular Reactivity

Endothelial function was assessed in proximal descending aorta sections as previously described [32]. The descending thoracic aorta was dissected and segmented (1.5–2 mm). Then, the arterial segments were placed in modified Krebs–Henseleit bicarbonate solution (KHS) (118 mmol L^−1^ NaCl, 4.75 mmol L^−1^ KCl, 25 mmol L^−1^ NaHCO3, 1.2 mmol L^−1^ MgSO4, 1.8 mmol L^−1^ CaCl2, 1 mmol L^−1^ KH2PO4, and 11 mmol L^−1^ glucose) and mounted in a wire myograph (Danish MyoTechnology^®^, Aarhus, Denmark) with a resting tension of 5 mN. After a period of 30 min, maximal contraction capacity was obtained by depolarization by 80 mmol L^−1^ KCl and the addition of the thromboxane receptor agonist 9,11-dideoxy-11α,9α-epoxymethanoprostaglandin F2α (U46619) at 0.003 mmol L^−1^. Endothelial functionality was assessed by vasodilation in response to acetylcholine (ACh 0.001 mmol L^−1^). After a 2 h washing period, vessels contracted to 80% of their maximum capacity with U46619 and were exposed to cumulative concentrations of ACh (0.001–10 mmol L^−1^). The Ach-induced relaxation was described as a percentage of the contraction with the thromboxaneA2 agonist U46619.

### 2.9. RT-qPCR

Total RNA was extracted from isolated kidneys (100 mg) using the AllPrep DNA/RNA/miRNA Universal Kit (Qiagen^®^, Hilden, Germany) according to the manufacturer’s protocol. An iScript^TM^ cDNA Synthesis Kit (Biorad^®^, Hercules, CA, USA) was used to obtain cDNA from 500 ng of total RNA per cDNA reaction, previously quantified with a Nanodrop ND-1000 spectrophotometer (ThermoFisher Scientific^®^, Waltham, MA, USA). For the real-time qPCR (RT-qPCR), 10 ng μL^−1^ of the pre-amplified product was mixed with 3.5 μL of nuclease-free water, 5 μL of TaqMan^TM^ Fast Advanced Master Mix (ThermoFisher Scientific^®^, Waltham, MA, USA), and 0.5 μL of TaqMan^TM^ Assay (Thermo Fisher Scientific^®^, Waltham, MA, USA). Real-time PCR analysis was performed using a LightCycler^®^ 480 (Roche Applied Science^®^, Upper Bavaria, Germany), and cycling conditions were as follows: UNG incubation hold at 50 °C for 2 min; polymerase activation hold at 95 °C for 2 min; 40 cycles of 3 s at 95 °C followed by 30 s at 60 °C. Changes in dnmt1 (Mm01151063_m1; AF036007.1) were measured (ThermoFisher Scientific^®^, Waltham, MA, USA). Data were normalized to the expression of the housekeeping gene β-actin (actb) (Mm00607939_s1; AK078935.1) (ThermoFisher Scientific^®^, Waltham, MA, USA) in each treatment, and a fold change was expressed relative to the basal levels of RNA from the naïve-SD control group. The delta-delta cycle threshold (ΔΔCt) method was used to calculate the fold expression levels for each target gene [33]. All reactions were performed in triplicate, and a negative control without cDNA was included. 

### 2.10. miRNAs Expression

MiRNAs were isolated from frozen kidneys using the AllPrep DNA/RNA/miRNA Universal Kit (Qiagen^®^, Hilden, Germany) according to the manufacturer’s instructions. Total RNA was quantified using a Nanodrop ND-1000 spectrophotometer (Thermo Fisher Scientific^®^, Waltham, MA, USA), and the TaqMan miRNA Assay (Applied Biosystem^®^, Waltham, MA, USA) (Table 1) was used to analyze the expression of miRNA-146a, miRNA-126, miRNA-23b, and miRNA-24-3p, as previously described by Budd et al. [34]. Specific cDNA for each miRNA studied was obtained using a TaqMan^TM^ miRNA Reverse Transcription Kit (ThermoFisher Scientific^®^, Waltham, MA, USA). In brief, a reaction mixture was made up of 3.58 μL of nuclease-free water, 0.75 μL of 10× Buffer, 0.094 μL of RNase inhibitor, 1.5 μL of RT primer, 0.075 μL of dNTPs, and 10 ng of total RNA; it was incubated for 30 min at 16 °C, then at 42 °C for 30 min, and 85 °C for 5 min, and then the reaction was terminated. Then, TaqMan^TM^ Universal PCR Master Mix II, No Amp Erase UNG (ThermoFisher Scientific^®^, Waltham, MA, USA) was used for the qRT-PCR assay in a reaction mix also containing 3.335 μL of nuclease-free water, 0.5 μL of TM primer, and 0.8 μL of cDNA. This mixture was then transferred to a 96-well plate and analyzed with a LightCycler^®^ 480 (Roche Applied Science^®^, Upper Bavaria, Germany). The reaction conditions were as follows: 2 min at 50 °C, 10 min at 95 °C, 40 cycles of 15 s at 95 °C and 1 min at 60 °C, and cooling to 4 °C. Standard optimization procedures were carried out to determine the most appropriate housekeeping gene for the study. Mam-mU6 (Thermo Fisher Scientific^®^, Waltham, MA, USA), an endogenous RNA housekeeping control, was selected to normalize the miRNA cycle threshold in each sample, and a fold change was expressed relative to the basal miRNA levels of the naïve-SD control group. To calculate the fold expression levels for all of the experiments, the delta-delta cycle threshold method was used. Each reaction was performed in triplicate with a negative control (cDNA not added).

### 2.11. Data and Statistical Analysis

The data and statistical analyses comply with the recommendations on experimental design and analysis in pharmacology. All of the Western blots and image data presented are representative of at least six animals.

All studies were designed to generate homogenous groups through randomization and blinded analysis. The group size is the number of independent values; the statistical analysis was carried out using these independent values. The number is stated in the figure legends. The data are expressed as the mean ± standard error of the mean (SEM) and were analyzed using GraphPad Prism Version 5.04 software (San Diego, CA, USA). Statistical significance was evaluated by one-way ANOVA for comparison between multiple groups with the appropriate post hoc test as described in each figure legend. *p* values of <0.05 were considered significant. Protein expression was normalized to the control to show the relative changes. Outliers were defined as values that exceeded the distance from the median value by 50%. When the mean values were normalized, this was declared within the figure legends.

## 3. Results

### 3.1. OLA-Supplemented Diet Reduced SLE Lymphoid Organs Weight in Pristane-Induced SLE BALB/c Mice

**Figure 1:**
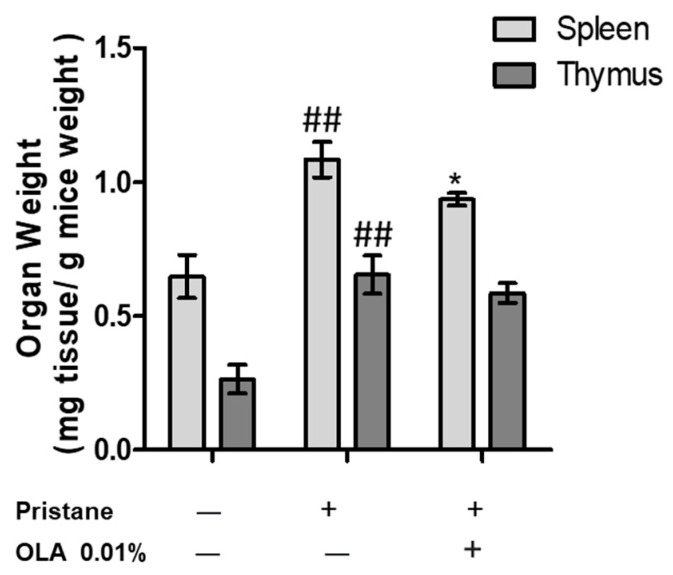
Immunomodulatory effect of an OLA-supplemented diet on the weight of the spleen and thymus organs 6 months after pristane induction. Data are expressed as the mean ± SEM (*n* = 10). One-way ANOVA followed by Tukey’s post hoc test results: ## *p* < 0.01 vs. naïve control group; * *p* < 0.05 vs. SD-pristane group.

The weights of the spleens and thymi were evaluated as markers of immune activity (Figure 1). As shown in Figure 1, the pristane-induced BALB/c mice presented higher thymus and spleen weights compared to the naïve control animals (## *p* < 0.01 vs. naïve group). Otherwise, dietary OLA treatment significantly ameliorated these manifestations in lymphoid organs, decreasing splenomegaly (* *p* < 0.05; vs. SD-pristane group). The changes observed in the thymus weight were not statistically significant.

### 3.2. OLA Dietary Treatment Counteracts Renal Abnormalities in Pristane-Induced SLE BALB/c Mice

**Figure 2:**
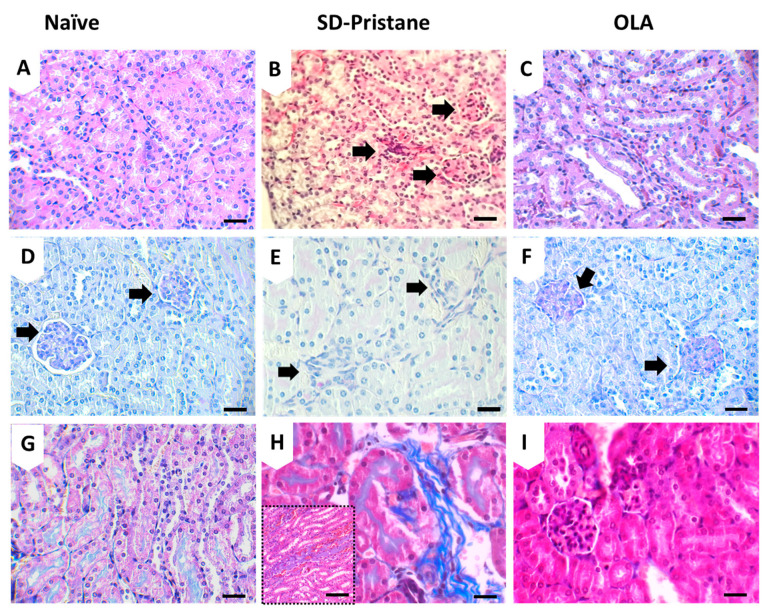
Histological effects of OLA dietary treatment on pristane-induced SLE mice. Representative images of (**A**–**C**) H&E staining; (**D**–**F**) PAS, and (**G**–**I**) MT stains from renal tissues from mice. Normal histology architecture in the cortex and medullary renal naïve mice with low stroma between renal tubules (**A**,**G**) and normal glomeruli in the cortex parenchyma (**D**, arrows). Compressed glomeruli in SD-Pristane mice (**B**, arrows), probably due to Bowman space proliferation with the partial sclerosis of glomerular tufts. Inflammatory cells occupying the medullary interstitium (**E**, arrows). Fibrotic tissue characterized by an abundance of collagen along with hemorrhagic signs identified in the medullary stroma (**H**). OLA-treated mice showed no histopathological changes either in the medullary or renal cortex. Normal distribution of renal tubules in medullary parenchyma (**C**), two glomeruli showing regular size and shape (**F**, arrows), and a renal cortex with no fibrosis or pathologic changes (**I**). Scale bar, 25 µm (**A**–**I**). Scale bar, 100 µm (**H** black-dotted squared).

Several histopathological kidney alterations were observed by H&E, MT, and PAS staining in the pristane group when compared to the control mice. The most relevant findings were the foci of inflammatory cells scattered throughout the cortex and medullary stroma (Figure 2E); frequent hemorrhagic areas so that renal tubules often contained red cell casts (Figure 2H); interstitial fibrosis (Figure 2H); and mild glomerular abnormalities with obliterated Bowman space compressing the glomerular tuft (Figure 2B). Moreover, signs of thyroidization in some renal tubules were identified using the PAS technique (Figure 2E) compared to kidneys from the naïve control group. After OLA administration, these phenomena were significantly reduced, and the kidneys showed a mostly normal histological appearance (Figure 2C, FyI). In fact, only a few inflammatory cells were identified, along with some renal glomeruli that were compressed due to the surrounding fibrotic signs. The percentage of renal injury exhibited by each group per item is summarized in Table 2.

**Figure 3:**
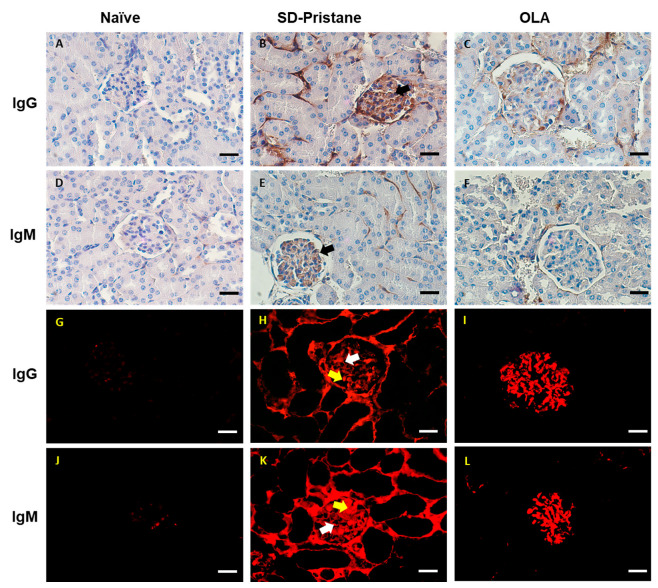
An OLA-supplemented diet reduced IgG and IgM deposits in the kidney tissue of pristane-induced SLE mice. Representative figures of (**A**–**F**) IHC and (**G**–**L**) IF staining of IgG or IgM in kidneys from pristane-induced SLE mice. (**A**,**D**,**G**,**J**) Absence of deposits of IgG and IgM in the naïve group. (**B**,**E**,**H**,**K**) Marked IgG and IgM deposits in the renal glomeruli of SD-pristane mice. (**B**,**E**, black arrows) Deposits in the capillary loops and mesangial cells and into the tubular interstices (extraglomerular nephritis). Deposits in capillary loops (**H**, white arrow) and mesangial deposits (**H**, yellow arrow) (mesangio—capillary glomerulonephritis). They were also accompanied by extraglomerular deposits. Mesangial glomerulonephritis (**K**, yellow arrow) also appeared with intracapillary deposits (**K**, white arrow) and tubular involvement. Scale bar, 25 µm.

The deposits of the IgG and IgM immunocomplexes play a critical role in the pathogenesis of lupus-induced nephritis [35]. Thus, we evaluated the presence of IgG and IgM immunocomplexes by IHC and IF in renal sections (Figure 3). In the naïve animals, no signs of nephritis were found, exhibiting negative immunostaining for IgG and IgM complexes (Figure 3A,D,G,J). Nevertheless, in SD-pristane mice, we observed pronounced deposits of IgG and IgM in all the structures that integrate the renal corpuscles (Figure 3B,E,H,K). 

In IHC, the immunocomplexes were deposited in the renal glomeruli; the deposits of igM were less abundant than those of IgG, as shown in Figure 3 (Figure 3B,E). In both cases, immunocomplexes can be seen in the capillary loops and in the mesangial cells (mesangio–capillary nephritis). In the tubular interstices, abundant deposits for IgG and a diffuse pattern for IgM were detected, leading to extra-glomerular nephritis. According to IHC, in IF, the deposits of IgG were clearly observed in the capillary loops and mesangial cells (Figure 3H) (mesangio-capillary glomerulonephritis). They were also accompanied by extraglomerular deposits. The presence of the IgM complexes showed mainly mesangial glomerulonephritis (Figure 3K) with intracapillary deposits and tubular involvement. Therefore, our results suggest a possible membranous LN in SD-pristane mice.

However, after dietary treatment with OLA, only a few mesangial deposits were identified in the renal glomeruli by IHC (Figure 3C,F); no extraglomerular deposits were detected. In IF, immunocomplexes were found in mesangial cells, with some subendothelial deposits having a granular appearance (Figure 3I,L). Nevertheless, no extraglomerular deposits were observed. Extraglomerular deposits are rarely present without deposits in the glomeruli, and the fact that we only detected glomeruli affectation can be understood as less involvement. These findings demonstrate the protective role of OLA in LN.

### 3.3. Effects of OLA Dietary Supplementation onCOX-2, iNOS, and mPGES-1 Protein Expression in Renal Tissue from Pristane-Induced SLE Mice

**Figure 4:**
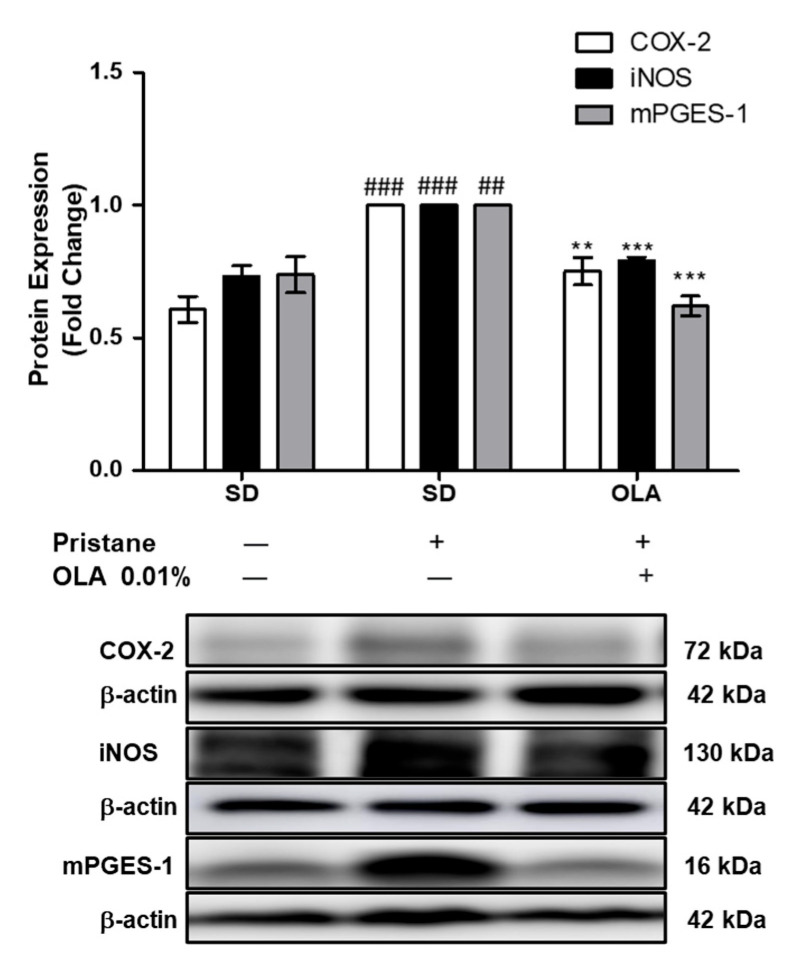
Dietary OLA administration reduced COX-2, iNOS, and mPGES-1 protein overexpression. Protein expression was measured in total kidney homogenates from mice. Densitometry was performed after normalization to the control (β-actin housekeeping gene). One-way ANOVA followed by Tukey’s post hoc test results: ## *p* < 0.01, ### *p* < 0.001 vs. naïve group; ** *p* < 0.01, *** *p* < 0.001 vs. SD-pristane group. Data were represented as the mean ± SEM (*n* = 6).

We evaluated the effects of OLA dietary treatment on COX-2, mPGES-1, and iNOS protein expression in kidney homogenates (Figure 4). The SD-pristane group showed a marked overexpression of COX-2, iNOS, and mPGES-1 proteins (## *p* < 0.01, ### *p* < 0.001 vs. naïve group). However, dietary supplementation with OLA significantly downregulated the expression of the proinflammatory enzymes COX-2, iNOS, and mPGES-1 to levels similar to those of the naïve control animals (** *p* < 0.01, *** *p* < 0.001 vs. SD-pristane group).

### 3.4. Effects of OLA Treatment on the Modulation of the MAPK, JAK/STAT, Nrf/HO-1, and Nf-kB Signaling Pathways in Pristane-Induced Mice

In order to further elucidate the possible molecular mechanisms involved in the immunomodulatory effects of an OLA-enriched diet, we evaluated the protein expression of several signaling pathways implicated in SLE pathogenesis: MAPKs, the Nrf-2/HO-1 axis, JAK3/STAT3 phosphorylation, and the NF-κB signaling pathways. 

**Figure 5:**
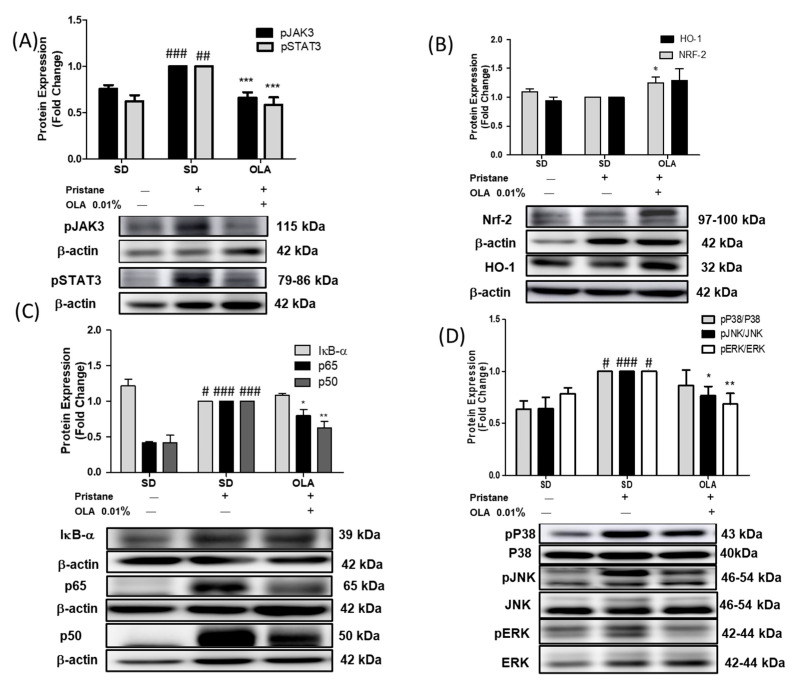
Role of the OLA dietary treatment in the regulation of SLE-related intracellular signaling pathways. Protein expressions of (**A**) phosphorylated JAK3/STAT-3; (**B**) the Nrf-2/HO-1 axis; (**C**) the IκB-α and p65/p50 nuclear subunits, and (**D**) phosphorylated P38, JNK, and ERK MAPKs were evaluated in renal homogenates from mice. Densitometry was performed after normalization to the control (JNK, ERK, p38, or β-actin). Data are represented as the means ± SEM (*n* = 6). One-way ANOVA followed by Tukey’s post hoc test results: # *p* < 0.05, ## *p* < 0.01, ### *p* < 0.001 vs. naïve group; * *p* < 0.05, ** *p* < 0.01, *** *p* < 0.001 significant difference versus SD-pristane group.

After 24 weeks of pristane injection, we observed a significant increase in the phosphorylation of JAK3 and STAT3 proteins in the SD-pristane group (Figure 5A) (## *p* < 0.01, ### *p* < 0.001 vs. naïve group). However, an OLA-enriched diet significantly downregulated the phosphorylation of both proteins (*** *p* < 0.001 vs. SD-pristane group). On the other hand, we detected a significant increase in Nrf-2 protein expression in the OLA-supplemented group (* *p* < 0.05 vs. SD-pristane group) (Figure 5B). This increase was also found in HO-1 protein expression, but it was not statistically significant.

Six months after the pristane injection, the translocation of the p65 and p50 proteins to the nucleus was significantly increased in the SD-pristane mice (### *p* < 0.001 vs. naïve group). Moreover, our data showed a significant degradation of the inhibitory protein IκB-α in SD-pristane mice (Figure 5C) (# *p* < 0.01 vs. naïve group). Interestingly, the OLA-enriched diet reduced p65 and p50 translocation to the nucleus compared to the SD-pristane group (* *p* < 0.05, ** *p* < 0.01 vs. SD-pristane group) (Figure 5C).

As Figure 5D shows, our results demonstrated an increase in the phosphorylation of p38, ERK, and JNK MAPKs in the SD-pristane mice in comparison to the naïve animals (# *p* < 0.05, ### *p* < 0.001 vs. naïve group). Nevertheless, an OLA-supplemented diet significantly decreased the phosphorylation of ERK and JNK MAPKs (* *p* < 0.05, ** *p* < 0.01 vs. SD-pristane group).

### 3.5. Nutritional OLA Supplementation Regulated Inflammasome Activation in Kidney Tissue from Pristane-Induced SLE Mice

**Figure 6:**
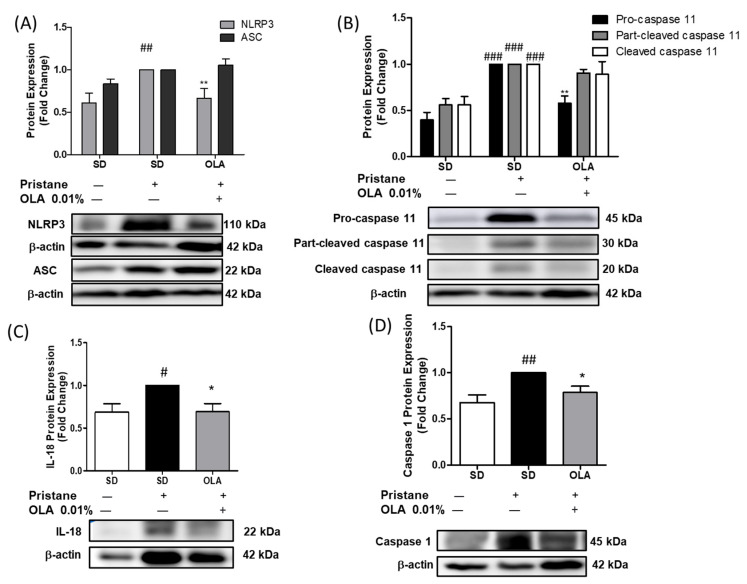
The inflammasome signaling pathway was downregulated by an OLA-supplemented diet. (**A**) NLRP3/ASC, (**B**) Caspase 11, (**C**) IL-18, and (**D**) Caspase 1. Protein expressions were analyzed by immunoblots in kidney lysates. Densitometry was performed following normalization to the control (β-actin housekeeping gene). Data are represented as the means ± SEM (*n* = 6). One-way analysis of variance (ANOVA), using Tukey–Kramer multiple comparisons test as post hoc test: # *p* < 0.05, ## *p* < 0.01, ### *p* < 0.001 vs. naïve group; * *p* < 0.05 ** *p* < 0.01; significant difference versus SD-pristane group.

We noticed a significant increase in NLRP3 expression, leading to caspase-1 activation and the maturation of IL-18 in SD-pristane animals (Figure 6) (# *p* < 0.05, ## *p* < 0.01 vs. naïve group). Nonetheless, OLA dietary supplementation significantly modulated the activation of the canonical inflammasome via the inhibition of NLRP3 and caspase-1 protein expression (Figure 6 A,D) (* *p* < 0.05, ** *p* < 0.01 vs. SD-pristane group). However, no statistically significant changes in ASC expression were observed after the OLA treatment. Moreover, the OLA-enriched diet also significantly regulated the non-canonical inflammasome, down-regulating pro-caspase 11 protein expression (Figure 6B) (** *p* < 0.01. SD-pristane group). Collectively, the levels of IL-18 were also reduced by an OLA-enriched diet (* *p* < 0.05 vs. SD-pristane group).

### 3.6. Effects of OLA Dietary Treatment on Vascular Function in the Thoracic Aortas from Pristane-Induced Mice

**Figure 7:**
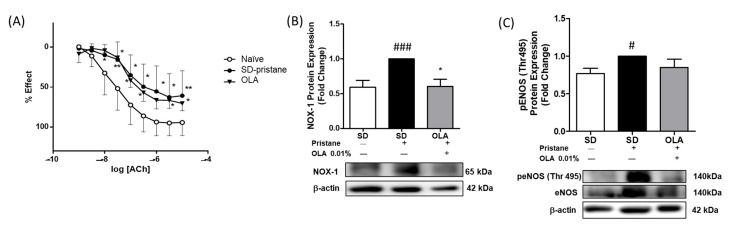
The OLA-supplemented diet enhanced redox-sensitive effectors and endothelial function in pristane-induced mice. (**B**) NOX-1 and (**C**) penos Thr495 protein expression quantification in homogenates of aortic rings from mice. Data are represented as the mean ± SEM (*n* = 6). β-Actin was used as a loading control. One-way ANOVA followed by Tukey’s post hoc test results: # *p* < 0.05, ### *p* < 0.001 vs. naïve group; * *p* < 0.05, vs. SD-pristane group. (**A**) Endothelial relaxation was induced by ACh (0.001–10 mM) in U46619 (0.003 mM) precontracted intact aorta rings from mice. The Ach-induced relaxant responses are expressed as a percentage of precontraction induced by U46619 (*n* = 7 per group). One-way ANOVA followed by Fisher’s LSD test results: * *p* < 0.05; ** *p* < 0.01 significant difference versus naïve control group (U46619), thromboxane receptor agonist 9,11-didesoxi-11α,9α-epoximetanoprostaglandina F2α.

Recent studies showed marked endothelial dysfunction in pristane-treated SLE mice [36,37]. Thus, we assessed the endothelium-dependent relaxation of ACh and deregulated contraction in murine aortic tissue. The aortic tissue in the SD-pristane group required significantly higher concentrations of ACh to relax compared to the naïve control group. However, a diet supplemented with OLA could not counteract the endothelial response after ACh exposure (Figure 7A).

Moreover, we studied endothelial dysfunction in aortic tissue from mice. The expression of NOX-1 and eNOS, redox-sensitive and oxidative stress-related pathways, was evaluated by Western blotting. As shown in Figure 7, we observed higher levels of expression of the NOX-1 protein in the SD-pristane mice compared with the naïve control group (### *p* < 0.001 vs. naïve group). In contrast, treatment with OLA-enriched diets normalized NOX-1 protein expression (* *p* < 0.05 vs. SD-pristane group). We also evaluated whether OLA was able to regulate the imbalance of peNOS (Thr495) phosphorylation induced by pristane in the mice’s aortas. Our results showed that the OLA diet was not able to significantly reduce the phosphorylation of Thr495 peNOS (Figure 7C).

### 3.7. An OLA-Supplemented Diet Regulated miRNAs and DNMT-1 Expression in Kidney Tissue

To assess the role of OLA dietary treatment in epigenetic modifications, we studied the expression of several miRNAs involved in SLE pathogenesis (miRNA-146a, miRNA-23b, miRNA-126, and miRNA-24-3p) and also the expression of DNMT-1 in renal tissue from pristane-induced mice. 

**Figure 8:**
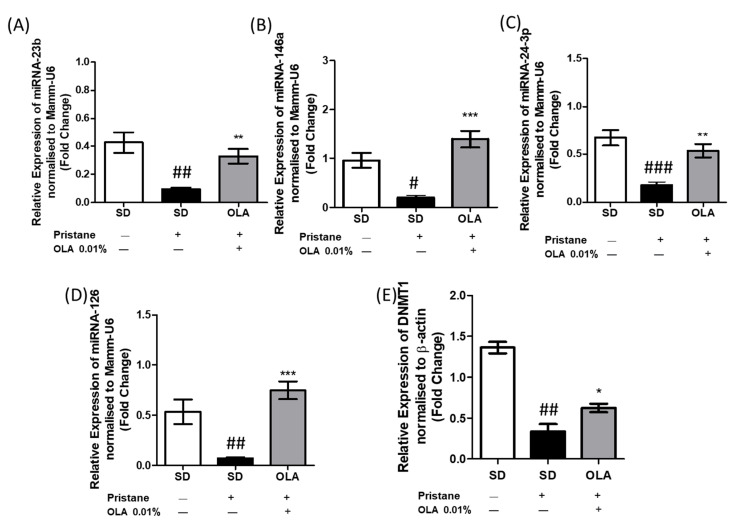
OLA dietary treatment modulated miRNAs and dnmt1 expression in pristane-induced nephritis. The relative renal expression of miRNAs: (**A**) miRNA-23b, (**B**) miRNA-146a, (**C**) miRNA-24-3p, and (**D**) miRNA-126, as well as (**E**) DNMT-1 mRNA from mice of the naïve, SD-pristane, and OLA-enriched diet groups, evaluated by RT-qPCR. Expression was normalized with MammU6 or β-actin, (*n* = 8). One-way ANOVA followed by Tukey’s post hoc test results: # *p* < 0.05, ## *p* < 0.01; ### *p* < 0.001 vs. naïve group; * *p* < 0,05, ** *p* < 0.01; *** *p* < 0.001, significant difference versus SD-pristane group.

miRNAs are small non-coding RNA molecules that modulate gene expression and play a crucial role in the pathogenesis of immune-inflammatory diseases; they have been suggested as biomarkers and therapeutic targets [38]. As Figure 8 shows, SD-pristane mice presented reduced expression of miRNA-24-3p, miRNA-146a, miRNA-23b, and miRNA-126 compared to mice from the naïve group (# *p* < 0.05, ## *p* < 0.01; ### *p* < 0.001 vs. naïve group). Nevertheless, after 6 months, the OLA-dietary treatment was able to counteract the miRNA overexpression and modulate the expression of miRNA-24-3p, miRNA-146a, miRNA-23b, and miRNA-126 in comparison to the SD-pristane group (* *p* < 0,05, ** *p* < 0.01; *** *p* < 0.001 vs. SD-pristane group), suggesting that OLA treatment could be supported by epigenetic mechanisms.

Furthermore, we evaluated the expression of DNMT-1. Previously, DNA hypomethylation has been detected in SLE [39]. In agreement with these data, we observed decreased DNMT-1 expression in SD-pristane, while an OLA-supplemented diet was able to restore its expression in renal tissue from pristane-induced mice.

## 4. Discussion

SLE is a chronic immune-inflammatory disease characterized by exacerbated B and T cell responses and the loss of immune self-tolerance [40]. Furthermore, SLE may be associated with comorbidities such as type 2 diabetes mellitus or cardiovascular diseases [41]. At present, SLE is a highly relevant autoimmune pathology due to the progressive degeneration of the quality of life of patients and the lack of an effective curative treatment. Consequently, these limitations have led to the search for other possible therapeutic strategies, such as nutritional therapy, which is recognized as an effective and safe tool for the management of chronic immune-mediated pathologies with limited drug treatments, such as SLE [42].

The pristane-induced lupus model is characterized by the presence of proteinuria, serositis, and glomerulonephritis. We observed splenomegaly and an increase in the thymus weight in SD-pristane-induced mice compared to the naïve group. Additionally, the histopathological kidney analysis showed hemorrhagic areas, interstitial fibrosis, and compressed glomeruli space with partial sclerosis of glomerular tufts in the pristane-treated mice, according to Song et al. [43]. The deposits of IgM and IgG observed indicated mesangio-capillary glomerulonephritis in pristane-induced renal tissue; our data are in agreement with previous reports that noticed similar histological changes [9,44]. However, dietary treatment with OLA reduced the lymphoid organ weight and improved renal tissue alterations, showing a normal cytoarchitecture with reduced IgG and IgM deposition.

COX-2 and mPGES-1, pro-inflammatory enzymes, are crucial factors in the production of PGE2, which is closely associated with autoimmune disease [45]. In renal tissue, COX-2 is inducible in interstitial cells, renal tubular epithelial cells, and mesangial cells. Furthermore, mPGES-1 is located in the macula densa, renal collecting ducts, and medullary interstitial cells [1,46]. On the other hand, it has been noted that the overexpression of iNOS and consequently high levels of NO are related to SLE development [47]. We found increased iNOS and COX-2/mPGES-1 axis protein expression in kidneys from SD-pristane mice, in line with previous reports [13,14]. However, after six months of OLA dietary administration, the expression of COX-2, iNOS, and mPGES-1 was significantly reduced in renal tissue. The ability of OLA to reduce the expression of these pro-inflammatory markers had previously been described in in vitro studies [18,19,20,21], but this is the first time that it has been evaluated in tissues from lupus mice, suggesting that dietary OLA may decrease the presence of inflammatory infiltrates and immuno-complexes in the kidneys of lupus mice by reducing the expression of COX-2, iNOS, and mPGES-1.

The transcription factor Nrf-2 is the main controller of redox-balanced homeostasis in cells, regulating antioxidant and anti-inflammatory responses [4]. Nrf-2 modulates the expression of antioxidant genes, such as HO-1. It has been described that kinases such as pERK and P38 and other signaling pathways such as NF-κB regulate Nrf-2 activation [48,49]. Currently, several reports elucidate the critical role of Nrf-2 regulation in SLE. In a previous work, Yoh et al. suggested that Nrf-2 deficiency plays a role in the development of SLE [50]. Moreover, the expression of the Nrf-2/HO-1 axis was downregulated in Balb/c mice [9,13]. In accordance with the studies mentioned above, our data showed suppressed Nrf-2 expression in pristane-induced SLE. However, the OLA-supplemented diet counteracts the effect of pristane by reestablishing Nrf-2 protein expression. These data are in agreement with those previously presented by Parzonko et al., who observed increased levels of Nrf-2 [51] in endothelial progenitor cells induced by OLA treatment. On the other hand, although OLA was unable to significantly increase HO-1 expression in the renal tissue of lupus mice, recent studies have noted an increase in HO-1 expression after OLA treatment [20]. These results suggest that the protective antioxidant effect of OLA in this experimental model of LES may be mediated through the regulation of Nrf-2. 

To elucidate the possible mechanisms by which OLA exerts its protective effect, the role of the MAPK pathway was investigated. The MAPK family participates in gene expression, cell differentiation, cell survival, and apoptosis; MAPKs contribute to the regulation of the immune-inflammatory response [52]. This serine/threonine protein kinase family mediates the activation of the NF-κB and NLRP3 inflammasome pathways [52] and regulates Nrf-2 expression, contributing to the autoimmune response [53]. Additionally, MAPKs induce the activation of the STAT3 protein by its phosphorylation on tyrosine residues, leading to the regulation of gene transcription [54]. In accordance with our previous results [7,9,13], we observed increased phosphorylation of p38, JNK, and ERK in kidneys from lupic animals. Moreover, our data are in agreement with those from MRL/lpr mice [35]. Thus, these findings may suggest a connection between the activation of MAPKs and SLE disease.

Additionally, the JAK/STAT signaling pathway has been described as relevant in the pathogenesis of immune-inflammatory diseases, including SLE [55]. Several JAK inhibitors are also used in patients with autoimmune rheumatic diseases. The blockage of the JAK/STAT axis could regulate the inhibition of the aberrant immune response [56]. Furthermore, Furumoto et al. identified a relationship between the JAK/STAT signaling pathway and vascular dysfunction in a murine lupus model [57]. Previously, it was demonstrated that OLA regulated pERK expression in an in vivo high-fat diet model and down-regulated pSTAT-3 phosphorylation in neuroblastoma cells [16,17]. In our study, the expression of the pSTAT-3/pJAK3 protein was upregulated in the kidneys of SD-pristane mice, corresponding to previous studies [9,13]. Interestingly, the phosphorylation of both MAPKs (JNK and ERK) and JAK3/STAT3 was modulated by an OLA-enriched diet in pristane-induced animals, confirming the role of the OLA-enriched diet in the regulation of the JAK/STAT and MAPK signaling pathways.

The key role of the NF-κB signaling pathway in SLE pathogenesis is well known; it controls the survival and proliferation of autoreactive T and B cells, but also regulates the inflammatory response by modulating the expression of pro-inflammatory mediators such as Th1/Th17 cytokines and the enzymes COX-2 and iNOs [58]. In addition, STAT-3 is related to NF-κB signaling through its ability to block IκB kinases (IKK), contributing to persistent NF-κB activation [59]. 

In its inactive state, NF-κB is locked in the cytoplasm by its inhibitor IκB. Nevertheless, under inflammatory conditions, IkB is phosphorylated by the IκB kinase (IKK) complex, leading to IκB degradation. As a consequence, the p65 and p50 subunits translocate to the nucleus, regulating the expression of several inflammatory mediators [5]. We observed decreased expression of IκB in the cytosol and increased translocation of p65 and p50 subunits to the nucleus in pristane-induced mouse kidneys, in agreement with previous work [5,35]. However, dietary OLA decreased the translocation of p65 and p50 to the nucleus, blocking NF-κB activation. Our data agree with previous studies, which noted that OLA treatment was able to reduce the DNA binding activity of the p65 NF-κB subunit and therefore the activation of the NF-κB pathway in TNF-α-stimulated adipocytes and LPS-activated THP1 cells [18]. Moreover, OLA reduced p65-NFκB overexpression in BV-2 microglia cells activated by LPS [21]. All of this, together with the fact that NF-κB inhibition leads to LN remission [60], presents OLA as a promising modulator of the NF-κB signaling pathway in LN development.

The NLRP3 inflammasome is a multimeric cytosolic complex that triggers pyroptosis, caspase-1 activation, and IL-1β and IL-18 maturation, leading to the inflammatory process. Moreover, NLRP3 inflammasome activation is induced by the NF-κB signaling pathway [61]. A growing number of studies have demonstrated the relationship between NLRP3 inflammasome activation and SLE [62]. Interestingly, inhibition of NLRP3 inflammasome activation ameliorates SLE disease in patients and SLE animal models [5,9,63]. In addition, increased levels of NLRP3 protein expression were detected in kidney biopsies from patients and were associated with SLE activity [63]. We observed significantly increased levels of NLRP3 and caspase 1 protein expression and IL-18 production in pristine-induced mice. However, an OLA-supplemented diet was able to modulate the canonical inflammasome pathway, in accordance with Gutierrez-Miranda et al., who observed downregulated expression of the NLRP3 protein in LPS-activated microglia cells after OLA exposure [21]. No changes were observed in ASC protein expression. Furthermore, caspase 11 integrates the non-canonical inflammasome signaling pathway; its maturation results in pyroptosis, potassium efflux, and the alternative activation of NLRP3. The non-canonical and canonical inflammasome pathways collaborate to induce tpro-IL-1 β and pro-IL-18 maturation [64]. In our study, an OLA-enriched diet was able to significantly reduce pristane-induced pro-caspase-11 expression and IL-18 maturation. This finding demonstrates for the first time that OLA can exert anti-inflammatory effects in pristane-induced SLE by regulating canonical and noncanonical inflammasome pathways.

Moreover, SLE patients have increased cardiovascular risk due to inflammation, endothelial dysfunction, and accelerated atherosclerosis, among other factors [65]. In recent years, cardiovascular events have constituted one of the principal causes of death in SLE patients [66]. Endothelial NO regulates the control of vascular tone, leukocyte migration, and blood clotting [67]. The increased production of ROS by NADPH oxidase such as NOX-1 and the subsequent reaction of ROS with NO have been described as key mechanisms of endothelial dysfunction [68]. In addition, altered NO production due to eNOS deficiency could produce impaired vascular reactivity [69]. We observed endothelial dysfunction in pristane-induced SLE mice, exhibiting impaired endothelium-dependent vasodilation, upregulated NOX-1 production, and imbalanced eNOS activation, according to other authors [9,36,68]. On the other hand, dietary OLA treatment significantly reduced NOX-1 protein expression. which has been shown to be a main factor in the development of cardiovascular dysfunction due to ROS overproduction [70]. However, despite these promising results, OLA was not able to regulate the impaired vascular relaxation response to the endothelium-dependent vasodilator ACh in SLE mice or the phosphorylation of pENOS (Thr 495), which could be observed after treatment with other secoiridoids of EVOO, such as oleocanthal [9].

The role of OLA in the regulation of key microRNAs for cancer has been described [25], but the effect of OLA on the regulation of microRNAs that influence the development of autoimmune diseases has not been studied. Recently, several preclinical and clinical studies have revealed the association between the development of LN and epigenetics, specifically the role of miRNAs. Interestingly, miR-146a has been presented as a protective factor against LN. Increased levels of miR-146a are related to the inhibition of pro-inflammatory cytokine production (IL-6, TNF-α, and IL-1β) and the expression of inflammatory mediators, leading to the survival of lupus-prone mice [71]. Moreover, Zhu et al. (2017) described the relationship between miR-146a expression and tumor necrosis factor receptor (TNFR)-associated factor 6 (TRAF6) in peripheral blood mononuclear cells (PBMC) in lupus patients. In fact, patients with increased TRAF6 expression and decreased miR-146a levels are more likely to experience the progression of LN [72]. We found down-regulated miR-146a expression in SD-pristane mice, according to Fu et al., 2019 [73]. However, dietary OLA treatment was able to counteract this effect, leading to miRNA-146a overexpression and improving LN. Our results confirmed a significantly ameliorated renal injury in animals fed with OLA dietary treatment, which was accompanied by an increase in miR-146a expression compared to pristane-induced mice.

Similarly, it has been suggested that miR-23b is related to the regulation of pro-inflammatory cytokines (TNF-α, IL-1β, and IL-17), which are crucial in inflammatory diseases such as RA and SLE. In addition, decreased expression of miR-23b has been described in autoimmune diseases [74]. According to these data, OLA counteracted the inhibition of miR-23b expression in pristane-induced mice by increasing miR-23b levels in renal tissue. Moreover, OLA treatment modulated TNF-α, IL-1β, and IL-17 production in murine spleen cells [20].

Furthermore, miR-24-3p is involved in immune disorders and inflammation. Oladejo et al. noticed that up-regulated miR-24-3p expression attenuates inflammation by inhibiting the NF-κB/MAPK signaling pathways that lead to TRAF6 blockage [75]. Moreover, MiR-24-3p acted as a cardioprotective factor, protecting against myocardial ischemia and reperfusion injury [76]. Our findings revealed lower levels of miRNA-24-3p expression in SD-pristane mice, which could be related to kidney damage. However, after 24 weeks of an OLA-supplemented diet, miR-24-3p expression had recovered to levels similar to those in naïve animals.

Recently, decreased levels of miR-126 have been found in plasma from patients with SLE, this could be crucial for the initiation and development of lupus by inhibiting the production of IFN-γ [77]. Accordingly, our data showed that the OLA-supplemented diet was able to restore miRNA-126 levels in kidney tissue and down-regulate IFN-γ production in murine splenocytes. In addition, miRNA-126 has been described as being related to DNMT-1 expression. DNA hypomethylation was observed in lymphocytes from lupus patients, this reduced DNA methylation is correlated with disease activity [78]. In agreement with the findings above mentioned, we observed decreased DNMT-1 expression in SD-pristane mice, which may lead to DNA hypomethylation. On the other hand, an OLA-supplemented diet significantly enhanced the production of DNMT-1. The overexpression of DNMT-1 suggests that OLA could modulate DNA hypomethylation by DNMT1 regulation, which is involved in SLE immunoinflammatory-related gene expression.

## 5. Conclusions

In conclusion, this study suggests the antioxidant, anti-inflammatory, and immunomodulatory effects of an OLA-supplemented diet in pristane-induced SLE mice. OLA ameliorated kidney damage by restoring renal cytoarchitecture and reducing the deposition of immune complexes. The mechanisms underlying these protective effects could be related to the modulation of Nrf-2 expression as well as the inhibition of signaling pathways including MAPKs, JAK/STAT, NF-κB, and inflammasome, which could possibly be correlated with the regulation of epigenetic marks on miRNAs and DNMT-1 expression. In addition, an OLA-enriched diet normalized NOX-1 overexpression in aortic tissue. This work introduces OLA as a promising epinutraceutical compound that may be useful in managing SLE. However, additional research is necessary to explore the beneficial effects of OLA, primarily to evaluate these effects in clinical trials.

## Figures and Tables

**Table 1:** miRNA expression studied using TaqMan miRNA Assays from Applied Biosystem.

MiRNA	TaqMan miRNA Assay Name	Assay ID
miR126	hsa-miR-126	002228
miR146a	hsa-miR-146a	000468
miR24-3p	hsa-miR24-3p	000402
miR23b	hsa-miR23b	000400
MammU6	U6 snRNA	001973

**Table 2:** Percentage of histopathological alterations per group.

	Hemorrhage	Inflammatory Cells	Glomeruli Alterations	Interstitial Fibrosis
Pristane	75%	80%	50%	80%
OLA	5%	30%	25%	5%

## Data Availability

Data are contained within the article.
